# Altered connectivity among the triple brain networks in patients with mild cognitive impairment: a source-based morphometry study with a large elderly population

**DOI:** 10.1007/s11604-025-01791-9

**Published:** 2025-06-18

**Authors:** Chihiro Yotsuya, Keita Watanabe, Sera Kasai, Yoshihito Umemura, Tomohiro Shintaku, Yuka Ishimoto, Miho Sasaki, Haruka Nagaya, Soichiro Tatsuo, Tatsuya Mikami, Yoshinori Tamada, Satoru Ide, Masahiko Tomiyama, Shingo Kakeda

**Affiliations:** 1 Department of Radiology, Graduate School of Medicine, Hirosaki University, 5 Zaifu-Cho, Hirosaki, Aomori 036-8562 Japan; 2 Department of Radiology, Kyoto Prefectural University of Medicine, 465 Kajiimachi, Jokyo-Ku, Kyoto, 602-8566 Japan; 3 Innovation Center for Health Promotion, Graduate School of Medicine, Hirosaki University, 5 Zaifu-Cho, Hirosaki, Aomori 036-8562 Japan; 4 Department of Medical Data Intelligence, Research Center for Health-Medical Data Science, Graduate School of Medicine, Hirosaki University, 5 Zaifu-cho, Hirosaki, Aomori 036-8562 Japan; 5 Department of Radiology, School of Medicine, University of Occupational and Environmental Health, 1-1, Iseigaoka, Yahatanishi-Ku Kitakyushu, Fukuoka, 807-8555 Japan; 6 Department of Neurology, Graduate School of Medicine, Hirosaki University, 5 Zaifu-cho, Hirosaki, Aomori, 036-8562 Japan

**Keywords:** Mild cognitive impairment, Source-based morphometry, Structural network, Brain networks, Magnetic resonance imaging

## Abstract

**Purpose:**

Previous research indicates brain network alterations in the default mode network (DMN), salience network (SN), and central-executive network (CEN) in individuals with mild cognitive impairment (MCI). However, replication has been inconsistent due to small, varied samples. We aimed to explore intra- and inter-networks alternations among DMN, SN, and CEN in individuals with MCI using multivariate source-based morphometry (SBM) with a larger, population-based sample.

**Materials and methods:**

This cross-sectional study included 1,997 participants (median age: 69 years; 61.9% female) who underwent three-dimensional (3D) T1-weighed imaging with 3 T magnetic resonance imaging (MRI). They were classified into 1,236 healthy controls (HC) and 761 individuals with MCI. SBM was used to extract triple brain networks as structural networks, and *Z*-scores were calculated. Intra-network comparisons of DMN, SN, and CEN between HC and MCI groups were conducted using logistic regression analysis. Inter-network comparisons among the triple brain networks were performed using structural equation modeling (SEM).

**Results:**

Connectivity (median *Z* score) of each network was lower in the MCI group than in the HC group: DMN (0.08 vs. − 0.12), SN (0.08 vs. − 0.23), and CEN (0.07 vs. − 0.06). Logistic regression showed significant association of SN connectivity with MCI (odds ratio 0.862, *p* < 0.05). SEM analysis revealed a significant group difference in the model where SN mediated input from CEN to DMN.

**Conclusion:**

We found altered network patterns in individuals with mild cognitive impairment, suggesting a transformation in network connectivity among DMN, SN, and CEN, particularly compensating for degraded SN connectivity.

## Introduction

Cognitive impairment in older adults is a growing social concern in many countries [1]. Mild cognitive impairment (MCI) is characterized by a more serious cognitive decline than normal aging and is associated with a high incidence of Alzheimer’s disease (AD) [2]. Early intervention for MCI is appropriate to prevent the progression of cognitive decline [3], which is crucial to delay the progression of MCI to AD. Therefore, the identification of individuals with MCI has been a persistent focus of research [4].

Resting-state functional magnetic resonance imaging (rs-fMRI) allows for the non-invasive detection of spontaneous neural activity at baseline. It is a useful tool to investigate the properties of functional brain networks, both locally and globally [5]. Many previous rs-fMRI studies have focused on the differences between individuals with MCI and healthy controls (HC) and have indicated altered patterns of brain networks related to cognitive decline. As cognition-related networks, default mode networks (DMNs), salience networks (SNs), and central-executive networks (CENs) are widely recognized [6–8].

A schematic review of rs-fMRI studies has indicated decreased connectivity in the DMN in individuals with MCI and AD [9]. Furthermore, studies have reported abnormal connections in the brains of patients with AD, not only in the DMN but also in the CEN and SN. Sorg et al. [10] reported reduced connectivity in the DMN and CEN hubs in individuals with MCI (*n* = 24). Meanwhile, Agosta et al. [11] reported decreased connectivity in the DMN among individuals with MCI (*n* = 12) and AD (*n* = 13) and increased mean connectivity in the CEN among patients with AD. Furthermore, Zhou et al. [12] reported increased functional connectivity in the SN of patients with AD (*n* = 12). Although these reports suggest the importance of investigating the roles of these three cognition-related networks in individuals with cognitive impairment, there have been no consistent findings. Furthermore, the DMN and CEN have consistently been reported to exhibit antagonistic activity in the resting state and during task performance in healthy individuals [13–15].

Interactions between these networks have been argued to be important for the effective maintenance of normal cognitive status [16–18]. In recent studies, MCI has been associated with disrupted interactions among the DMN, SN, and CEN [8]. Thus, connections among these networks have recently been proposed as a triple network model [17–20] to understand altered network patterns in cognitive impairments. The triple network model suggests that the SN dynamically controls network interactions between the DMN and CEN and that the core role of the SN is to initiate network-mediated dynamic interactions between the CEN and DMN [8, 19, 21]. To the best of our knowledge, population-based studies with large sample sizes have not been conducted to evaluate connectivity among the triple networks in MCI.

Recently, a source-based morphometry (SBM) technique utilizing 3D T1-weighted imaging (T1WI) data was introduced. This multivariate approach pools information across different voxels and identifies unpredictable patterns thereof [22, 23]. SBM applies independent component analysis (ICA) to segmented images, arranges voxels into sets containing similar information [22], and acquires common morphological features among individuals at the brain network level. Importantly, the underlying hypothesis of morphological network analysis is that brain regions exhibiting similar structural characteristics—such as gray matter volume or cortical thickness—may be developmentally, genetically, or functionally related. This morphological covariance is thought to reflect shared neurobiological processes, suggesting that these regions could be interconnected at a systems level [24]. Three-dimensional (3D) T1WI has also been included in the standard brain magnetic resonance imaging (MRI) protocol. Conversely, functional MRI has been widely used for research, not in clinical situations. Therefore, the SBM method using 3D T1WI data is convenient to identify novel networks and may be a reasonable tool for brain MRI studies in larger populations. Moreover, structural MRI imaging, compared to rs-fMRI, has the advantage of high spatial resolution.

We reviewed data from a population-based prospective study on dementia in a large population of older Japanese individuals (The Iki-Iki Health Promotion Project: Iki-Iki study) to acquire and analyze the brain networks using SBM. We then aimed to assess the network connectivity of individuals with MCI compared with cognitively normal older adults as HC among the three cognition-related networks by applying SBM. Furthermore, we evaluated the differences in network interactions among the DMN, SN, and CEN between HC and individuals with MCI.

## Materials and methods

### Study population and design

This study was conducted in accordance with the ethical guidelines of the Declaration of Helsinki. The use of data from The Iki-Iki study was approved by the Ethics Committee. Written informed consent was obtained from all participants. The Iki-Iki study was established in 2016 as a population-based 12-year prospective study of cerebrovascular and cardiovascular disease and dementia in an older Japanese population from the Iwaki area of Hirosaki City, Aomori Prefecture, Japan. This is one study field of the Japan Prospective Studies Collaboration for Aging and Dementia (JPSC-AD) that is a multisite, population-based prospective cohort study of dementia [25, 26]. A total of 2,390 participants aged > 64 years participated in the screening surveys in 2016 and 2017, and 2,226 (93.1%) participants underwent brain MRI. We excluded 50 participants with image distortions (seven with metal artifacts, 13 with excessive motion artifacts, and 30 for whom brain volume or structural covariance intra-networks could not be measured accurately for various reasons) and 43 participants without available MRI data (T1-weighted images). Of the 2,133 participants, 761 (35.8%) with Mini-Mental State Examination (MMSE) scores of 24–27 were defined as MCI [27–29]. A total of 1,236 (57.9%) participants with MMSE scores of 28–30 were defined as HC, and 136 (6.3%) participants with ≤ 23 MMSE were excluded due to significant cognitive decline [28]. Thus, 1,997 participants (761 with MCI and 1,236 HC) were enrolled in this study (Table 1).

**Table 1:** Clinical characteristics of participants

	HC	MCI	*P* Value
	(*N* = 1236)	(*N* = 761)	
Age, median (IQR)	68 (64–80)	70 (64–80)	< 0.01
Sex: male/female	449/787	312/449	0.04
Education: High school/Junior high school	1091/145	572/189	< 0.01
MMSE, mean (SD)	29.26 (0.77)	25.79 (1.05)	< 0.01
DMN, median (95% CI)	0.08 (− 0.73, 0.88)	− 0.12 (− 0.89, 0.63)	0.18
SN, median (95% CI)	0.08 (− 0.77, 1.03)	− 0.23 (− 1.10, 0.69)	< 0.01
CEN, median (95% CI)	0.07 (− 0.91, 1.08)	− 0.06 (− 1.08, 0.89)	0.10
HP/ICV ratio, mean (SD)	0.0055 (0.00049)	0.0054 (0.00052)	< 0.01
GMV/ICV ratio, mean (SD)	0.42 (0.023)	0.42 (0.024)	< 0.01
WMV/ICV ratio, mean (SD)	0.32 (0.019)	0.32 (0.020)	< 0.01

*MCI* mild cognitive impairment, *HC* healthy controls, *DMN* default mode network, *SN* salience network, *CEN* central-executive network, *HIP* hippocampus, *ICV* intracranial volume, *GMV* gray matter volume, *WMV* white matter volume, *CI* confidence coefficient, *SD* standard deviation, *IQR* interquartile range, *MMSE* Mini-Mental State Examination

### MRI

All brain MRI data were obtained using the same protocol on a single 3 T MRI scanner (Signa EXCITE 3 T; GE Healthcare, Waukesha, WI) with an eight-channel brain phased-array coil. Original T1-weighted images were obtained in the steady state using a 3D fast spoiled gradient recalled sequence with the following parameters: repetition time, 10 ms; echo time, 4.1 ms; inversion time, 700 ms; flip angle, 10; field of view, 26 cm; section thickness, 1.2 mm; resolution, 1.0 × 1.0 × 1.2 mm; Number of Excitations, 1; and imaging time, 3 min 20 s.

### Image processing for voxel-based morphometry

Image preprocessing was identical to that used for classical voxel-based morphometry analyses. A fully automatic technique for the computational analysis of differences in regional brain volume throughout the brain was conducted using SPM12 software (Institute of Neurology, London, UK) [30, 31]. The 3D fast spoiled gradient-echo images in the native space were spatially normalized; segmented into gray matter (GM), white matter (WM), and cerebrospinal fluid images; and modulated using the Diffeomorphic Anatomical Registration Through Exponential Lie Algebra (DARTEL) toolbox in SPM12 [32]. DARTEL was devised as an alternative method for normalization in the SPM package [30]. To preserve the GM and WM volumes within each voxel, we modulated the images using Jacobean determinants derived from spatial normalization using DARTEL. The resulting modulated GM images were smoothed using an 8-mm full-width-at-half-maximum Gaussian kernel. The analysis was conducted using the CAT12 toolbox (C. Gaser, Structural Brain Mapping Group, Jena University Hospital, Jena, Germany; http://dbm.neuro.uni-jena.de/cat/), which is implemented in SPM12 (Wellcome Trust Center for Neuroimaging, London, UK; http://www.fil.ion.ucl.ac.uk/spm/software/spm12/) [30, 31]. Intracranial volume (ICV), total gray matter volume (GMV), total white matter volume (WMV), and bilateral hippocampal volume (HV) were calculated using the 40-brain LPBA40 atlas [33]. GMV/ICV, WMV/ICV, and HV/ICV ratios were calculated. The HV/ICV ratio is used as an indicator of hippocampal atrophy.

### Image processing for SBM

SBM is a multivariate technique that utilizes ICA [22] and considers information across different voxels and identifies unpredicted, naturally occurring patterns of covariance across brain regions. The GIFT toolbox (http://icatb.sourceforge.net) was used for image processing in the SBM analysis [22]. The minimum description length principle was used to estimate the number of independent components, identifying 147 reliable independent components (GM structural networks). We performed ICAs using a neural network algorithm (Infomax). Through ICAs, we attempted to minimize the mutual information of network outputs to identify natural groupings and maximally independent sources [34]. ICAs were repeated 20 times in ICASSO (http://research.ics.aalto.fi/ica/icasso/), and the obtained components were clustered to confirm consistency and reliability. Consequently, we selected a matrix in which 1,997 rows represented 1,997 subjects (1,236 HC and 761 MCI), and each column represented a voxel. This matrix was decomposed into two matrixes using ICA, the “mixing matrix” and the “source matrix.” The first matrix (“mixing matrix”) comprised one subject per row and IC per column and included loading coefficients that indicated the contribution of each structural component to the participants. Thus, information on the relationship between each participant and each component was included in the mixing matrix. Thus, the loading coefficients revealed the contribution of each participant to the specific brain network components. The second matrix was called the “source matrix” and prescribed the relationship between the independent components and the voxels. The loading coefficients were transformed into *Z*-scores using Fisher’s *z*-transformation. We visualized the information gained from the independent components. For visualization, the source matrix was reshaped back to a 3D image and scaled to unit standard deviations (*Z* maps); the threshold was set at *Z* > 2.0.

**Fig. 1:**
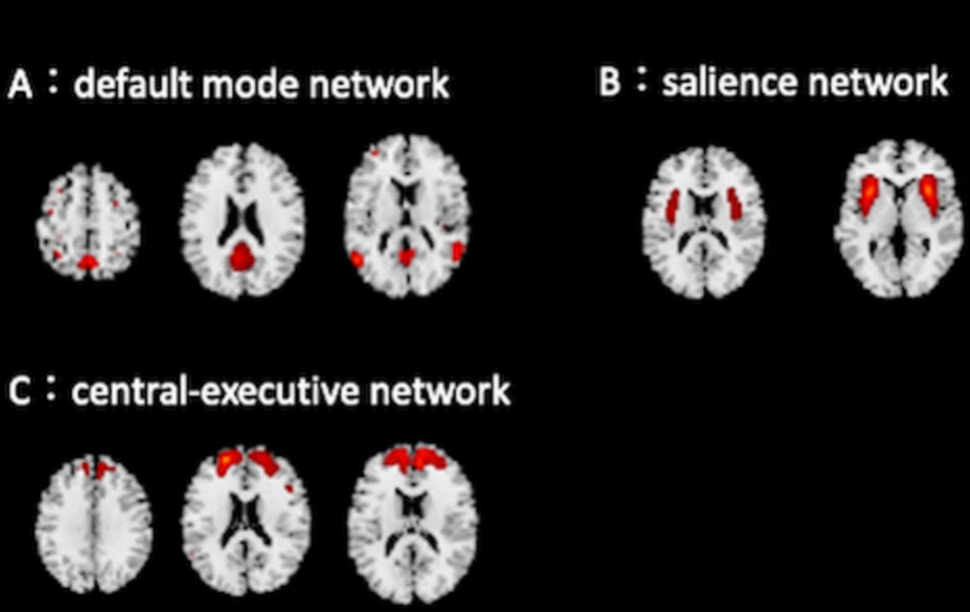
The main images of each network. (**A**) default mode network (DMN), (**B**) salience network (SN), and (**C**) central-executive network (CEN)

As a result, ICA generated 147 independent components (GM structural networks). From these components, two experienced neuroradiologists (S.K. with 24 years of experience, and K.W. with 12 years of experience) identified the three key networks: the DMN, SN, and CEN (Fig. 1). This identification was performed by visually inspecting each component against established patterns documented in prior research, specifically the atlases of brain networks tailored for an elderly cohort [35]. These atlases provide detailed anatomical regions typically associated with these networks, aiding in accurate identification; DMN: We selected components that prominently included the posterior cingulate cortex and the angular gyrus, which are critical regions of the DMN, SN: Components that contained the anterior insula were chosen, as this area is central to the SN, CEN: We identified components that involved the dorsolateral prefrontal cortex, a key region in the CEN. These selections were made by consensus between the two neuroradiologists.

The *Z*-scores in SBM allowed us to identify the sources that exhibited differences between the HC and MCI groups. Multivariable linear regression was used to investigate the association between cognitive decline and the loading coefficients (*Z*-scores) for each component. Regression coefficients within groups were determined using dummy variables for HC and MCI as reference groups. The analyses were adjusted for age, sex, and education level (below junior high school, higher than high school graduate, or equivalent).

### Statistical analyses

All statistical analyses were performed using EZR (Saitama Medical Center, Jichi Medical University, Saitama, Japan) [36], and statistical significance was set at *p* < 0.05. The clinical characteristics of the participants within the HC and MCI groups were compared using logistic regression analysis, Welch’s *t*-test, and Pearson’s chi-square test, as appropriate. Nominal variables are expressed as percentages and continuous variables as mean ± standard deviation, median (interquartile range), or range based on distribution. In the analysis of the loading coefficients calculated from the SBM for each component, we compared the two groups with the loading coefficients, as each participant had a loading coefficient for each component.

**Fig. 2:**
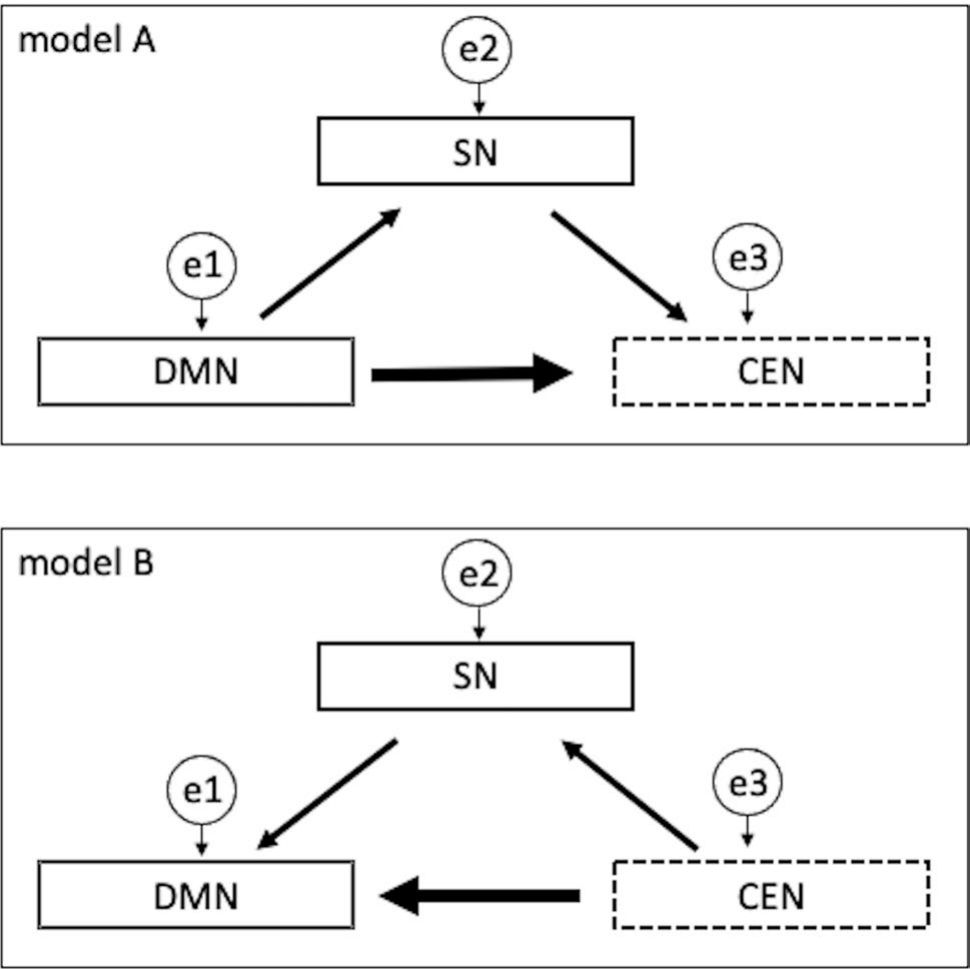
Path diagrams of the network model. Two path diagrams were constructed using a model in which the SN coordinated communication between the DMN and CEN. Model A: This diagram illustrates a network model assuming a direct connection from the Default Mode Network (DMN) to the Central-Executive Network (CEN) and an indirect connection mediated by the Salience Network (SN). The error variables indicate unexplained variance in each network in Model A. Model B: This diagram depicts a network model where the CEN has direct connections to the DMN, with the SN mediating additional indirect connections. Similar to Model A, error variables capture the unexplained variance in each network in Model B

Using SPSS AMOS (version 27.0; SPSS Inc., Chicago, IL) for structural equation modeling (SEM) analysis, we examined the role of the SN in mediating communication between the DMN and CEN in the HC and MCI groups. The constructed model posited that the SN coordinates communicate between the DMN and CEN [21]. To compare and examine the interactions among these three networks, we created two path diagrams based on a previous study [8] (Fig. 2). First, we created Model A in which the SN mediated network input from the DMN to the CEN. The other model (Model B) assumes that the SN mediates network input from the CEN to the DMN. Path diagrams were created for both models in the HC and MCI groups, and path coefficients were obtained. In the path diagram of Model A, the networks from the DMN to the CEN were compared by setting direct and indirect routes. The direct path reflected the input from the DMN to the CEN. Indirect pathways are models in which the SN intervenes in the network input from the DMN to the CEN. The error variable setting represented the variation left unexplained by the explanatory variables of the path model. In contrast, in the path diagram of Model B, we compared the network inputs from the CEN to the DMN by setting direct and indirect paths. The direct path reflected the input from the CEN to the DMN. An indirect pathway is a model in which the SN intervenes in the network input from the CEN to the DMN. In Model B, the error variable setting represented the variation left unexplained by the explanatory variables of the path model.

Models A and B were subjected to an SEM analysis. Several model fit indices should be considered to appropriately interpret the model. The model’s goodness of fit was tested using the Goodness of Fit Index (GFI) and the comparative fit index (CFI). When the GFI and CFI were greater than 0.95, they were considered good indices [37]. Briefly, the GFI is an absolute fit measure that reflects the proportion of variance explained by the model. In contrast, the CFI compares the fit of the proposed model to an independent baseline model, adjusting for model complexity. Both indices help assess model suitability but from different perspectives. In each model, path coefficients were gained, and a chi-square difference test was performed to evaluate differences in path coefficients between the HC group and the MCI group.

## Results

Table 1 presents the demographic characteristics of the participants. There were significant differences in the age, sex, and years of education between the HC and MCI groups. Connectivity across the various networks was generally diminished in the MCI group compared to the HC group. Median *Z*-scores for the DMN were 0.08 in the HC group versus − 0.12 in the MCI group; for the SN, 0.08 versus − 0.23; and for the CEN, 0.07 versus − 0.06 (Table 1).

Logistic regression analysis showed that SN connectivity (Model 2 in Table 2) was a significant independent predictor of MCI (*p* < 0.01) after adjusting for potential confounders (age, sex, and education level), but not DMN or CEN connectivity (Models 1 and 3 in Table 2). The GMV/ICV, WMV/ICV, and HV/ICV ratios were not significant independent predictors of MCI after adjustment (Models 4, 5, and 6 in Table 2), although the MCI group showed significantly reduced GMV/ICV, WMV/ICV, and HV/ICV ratios compared to the HC group (Table 1).

**Table 2:** Multiple regression analysis

Predictor	Model 1		Model 2		Model 3		Model 4		Model 5		Model 6	
	Odds ratio (95% CI)	*p*	Odds ratio (95% CI)	*p*	Odds ratio (95% CI)	*p*	Odds ratio (95% CI)	*p*	Odds ratio (95% CI)	p	Odds Ratio (95% CI)	p
Age	1.04 (1.02, 1.07)	< 0.01	1.04 (1.01, 1.06)	< 0.01	1.05 (1.02, 1.07)	< 0.01	0.998181 (0.997978, 0.998384)	< 0.01	0.997913 (0.997728, 0.998097)	< 0.01	0.999952 (0.999948, 0.999957)	< 0.01
Sex	0.796 (0.658, 0.963)	0.02	0.725 (0.595, 0.884)	< 0.01	0.791 (0.654, 0.958)	0.02	1.023310 (1.021518, 1.025048)	< 0.01	1.003676 (1.002128, 1.005226)	< 0.01	1.000312 (1.000273, 1.000351)	< 0.01
Education [High School Graduate]	1.28 (1.03, 1.59)	0.02	1.27 (1.02, 1.57)	0.03	1.29 (1.04, 1.60)	0.02	1.002779 (1.000902, 1.004659)	< 0.01	1.000586 (0.998883, 1.002293)	0.50	1.000034 (0.999990, 1.000077)	0.13
Education [below junior high school]	2.69 (2.03, 3.57)	< 0.01	2.64 (1.99, 3.51)	< 0.01	2.70 (2.03, 3.58)	< 0.01	1.005155 (1.002561, 1.007757)	< 0.01	1.000778 (0.998429, 1.003133)	0.52	1.000066 (1.000006, 1.000126)	0.03
DMN	0.93 (0.865, 1.03)	0.18										
SN			0.862 (0.80, 0.929)	< 0.01								
CEN					0.948 (0.89, 1.01)	0.10						
GMV / ICV							0.999229 (0.997504, 1.000957)	0.38				
WMV / ICV									0.999864 (0.998293, 1.001437)	0.87		
HV / ICV											0.999963 (0.999923, 1.000003)	0.07

*MCI* mild cognitive impairment, *DMN* default mode network, *SN* salience network, *CEN* central-executive network, *ICV* intracranial volume, *GMV* gray matter volume, *WMV* white matter volume, *HV* hippocampus volume, *CI* confidence coefficient

**Fig. 3:**
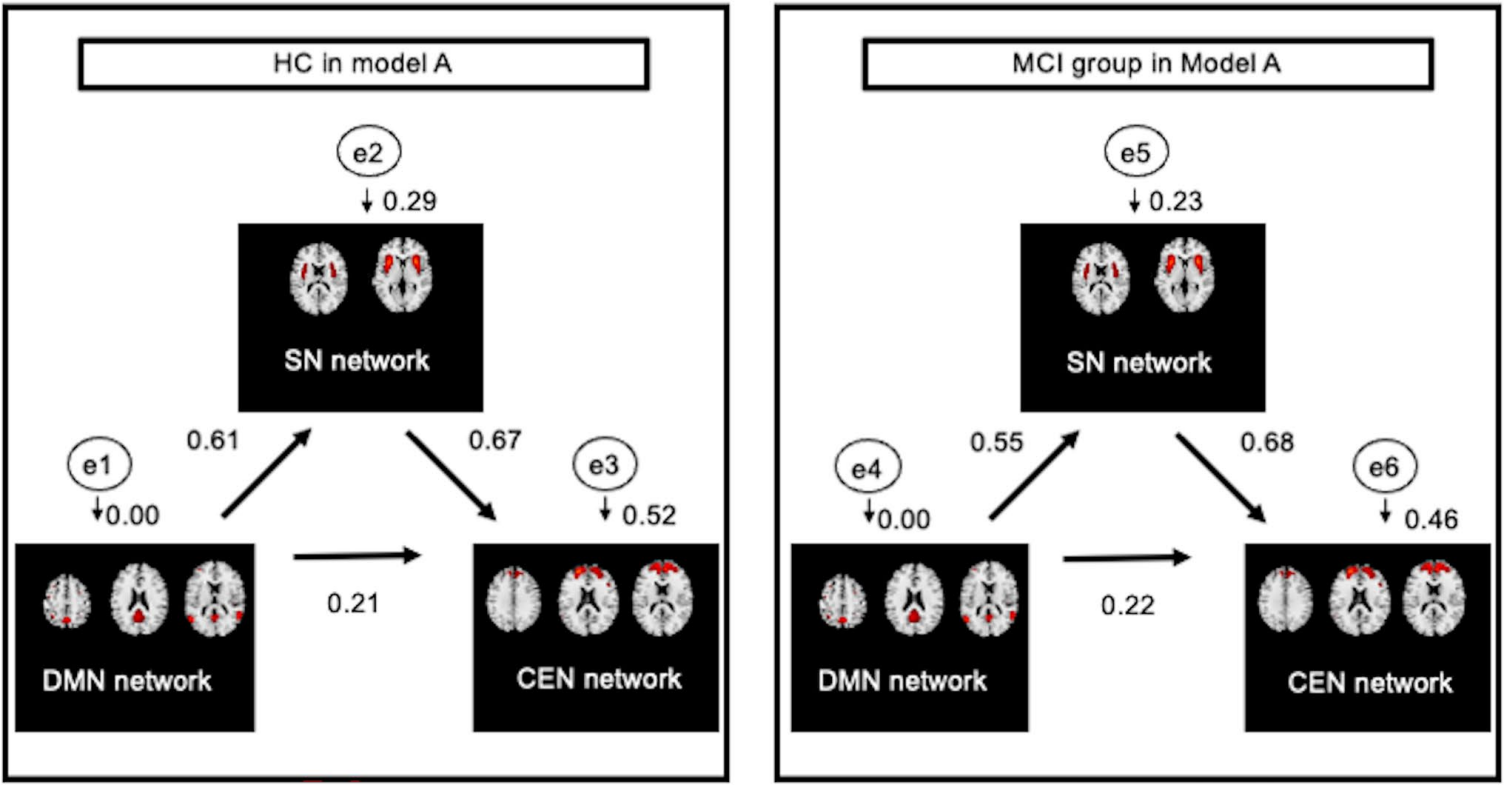
Path analysis in Model A showing a comparison of path coefficients between HC and MCI regarding networks from DMN to CEN. This path model shows that the SN inter-events with the connection from the DMN to the CEN. Each number near the arrows represents a path coefficient indicating the strength and direction of each connection. Here, e1 through e6 mean error variable. A small number represents the square of multiple correlation coefficients (*R*^2^) for each error variable. No significant differences were observed between the HC and MCI groups. *HC* healthy controls, *MCI* mild cognitive impairment, *DMN* default mode network, *SN* salience network, *CEN* central-executive network

**Fig. 4:**
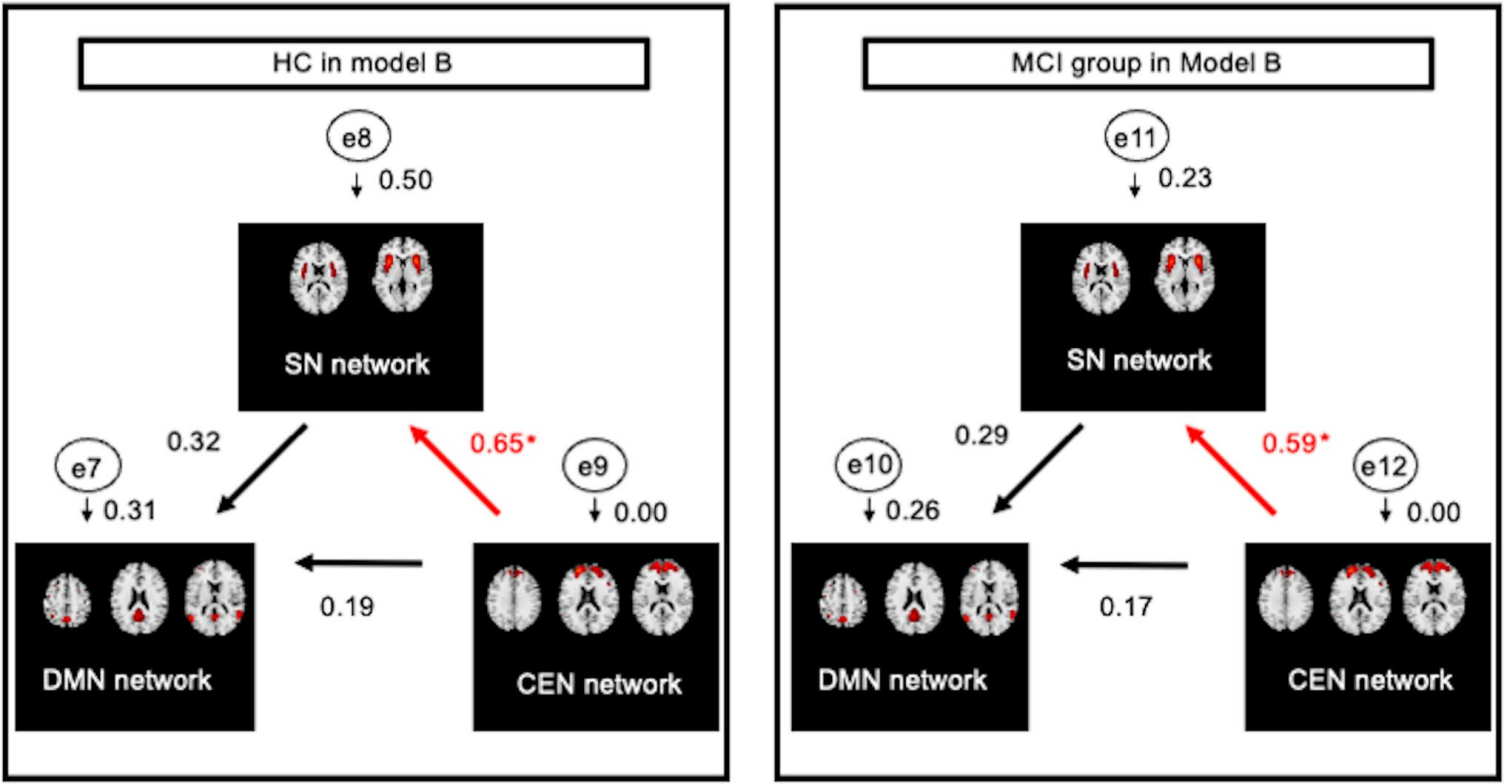
Path analysis in Model B showing a comparison of path coefficients between HC and individuals with MCI regarding networks from CEN to DMN. This path model shows that the SN interacts with the connections from the CEN to the DMN. Each number near the arrows represents a path coefficient. Here, e7 through e12 mean error variable. A small number represents the square of multiple correlation coefficients (*R*^2^) for each error variable. Significant differences in the network from the CEN to the SN were observed between the HC group and MCI group (*p = 0.01). *HC* healthy controls, *MCI* mild cognitive impairment, *DMN* default mode network, *SN* salience network, *CEN* central-executive network

In the SEM analysis, the results of testing the goodness of fit of Models A and B both showed GFI = 1.00, and CFI = 1.00. In both models, the goodness of fit indices were acceptable. While Model A (network model from DMN to CEN via the SN) did not show any significant differences between the HC and MCI group (Fig. 3), Model B (network model from CEN to DMN via the SN) showed notable disparities between the HC group and MCI group (Fig. 4) (*p* < 0.05).

## Discussion

Our multiple regression analysis of the brain networks showed that only SN connectivity was a significant independent predictor of MCI, after adjusting for potential confounders. The SEM analysis revealed a significant difference between the HC and MCI groups in a model in which the SN mediated network input from the CEN to the DMN. To our knowledge, this is the first population-based study with large sample sizes to demonstrate brain network alterations in individuals with MCI.

As for brain networks in cognitive impairment, although most studies have focused on DMN, recent studies showed that cognitive abilities affect not only DMN but also other networks [6, 7]. Furthermore, Onoda et al. demonstrated that disruption of the SN is associated with cognitive decline in elderly individuals [38]. Brier et al. found reduced connectivity in DMN and SN of individuals with MCI as well as patients with AD [39], and He et al. found that both the structural and functional organization of the SN was impaired in individuals with MCI and AD [40]. These results are consistent with those of the present study.

We found a significant difference between the HC and MCI groups in a model where the SN intermediates the inter-network from the CEN to the DMN, suggesting altered SN function between the DMN and CEN inter-network connectivity. The DMN has been shown to be more internally activated during self-monitoring, directed cognitive activities, and social functions. The CEN is often activated for externally directed higher-order cognitive functions, such as attention, decision-making, and working memory [16, 18]. The DMN and CEN are known to interact competitively during cognitive information processing [14, 41], and the SN drives switching between the DMN and CEN [17, 21, 42]. Chand et al. [8] showed that the disruption of SN control over the DMN and CEN provides a neuronal basis for cognitive decline and may be a candidate neuroimaging biomarker for cognitive impairment. Moreover, Li et al. [43] showed abnormal SN function at intra-network connectivity in patients with AD and late MCI, particularly SN-DMN and SN-CEN connectivity. These findings support our results that the SN plays a key role in mediating networks between the CEN and DMN and that there is a significant difference between HC and individuals with MCI.

Brain atrophy, as measured using MRI, is a remarkable sign of neurodegeneration, and different morphological analysis methods have been used to investigate subtle structural alterations in individuals with MCI [44]. However, improvements are still needed for the accurate diagnosis of MCI stages. In this study, we found no significant differences in brain volume measurements (WMV, GMV, and HV) between the HC and MCI groups using logistic regression analysis. The SN connectivity of the MCI group with SBM was significantly lower than that of the HC group. This suggests that the SBM has great potential to provide prognostic information for cognitive decline. Therefore, further studies are needed to clarify whether altered connectivity might precede volume changes in the brain parenchyma and whether altered connectivity and reduced brain volumes may reflect different and complementary information for MCI pathology.

There are many reports on brain networks in cognitive dysfunction using rs-fMRI. However, we used structural MRI to investigate brain networks and their interactions. Regarding the DMN, our logistic regression analysis did not show a significant difference in the DMN between the HC and MCI groups. Previous studies established that DMN activity is impaired in individuals with MCI and AD [5, 39]. However, a more recent systematic review of 57 MCI vs. HC comparisons suggested substantial inconsistency in patterns of altered resting DMN connectivity. Nine studies showed no group difference, eight studies showed MCI > HC in DMN connectivity, 22 studies showed HC > MCI across the brain, and 18 studies showed regionally mixed directions of effect [45]. Our study has several strengths compared to previous rs-fMRI studies. Our community-based design and large sample size reduced sampling bias and the effects of individual variability and permitted a detailed evaluation of confounding factors. Our SBM analysis was performed using 3D T1WI, which has been widely used for MR examinations in clinical situations. Thus, this method may be useful for brain MRI studies in larger populations. Additionally, this study was conducted using images captured on the same 3 T MRI scanner with a consistent protocol. Previous analyses have suggested that volume measurements across platforms (vendors, MRI sequences, and scanner upgrades) introduce a difference bias [46]. By using the same MRI scanner and protocol, this bias could be avoided.

In this study, we conducted network research using structural MRI rather than resting-state functional MRI (rs-fMRI). Traditionally, most network studies utilize rs-fMRI, a technique that captures a series of 3D brain images over time to analyze temporal correlations between BOLD signal fluctuations in different brain regions during rest. This method is effective in reflecting neural synchrony and transient brain states. However, our approach using SBM examines structural correlations by analyzing the covariance of differences from mean images across multiple scans instead of relying on temporal information. This approach allows us to identify covarying structural patterns across individuals, which may infer underlying network connections based on brain morphology. Unlike the dynamic interactions observed in rs-fMRI, the use of structural networks through SBM provides insights into long-term formations within the brain architecture. While structural network analysis with SBM is not identical to the functional connectivity measured by rs-fMRI, previous studies have shown a direct association between functional and structural covariance networks across the entire brain, suggesting a complementary relationship between these methods [47]. Furthermore, studies have reported the extraction of well-known networks such as the DMN, CEN, and SN using SBM [48], as demonstrated in the current study. These findings indicate that SBM can effectively reveal key networks and may serve as a valuable tool, particularly when rs-fMRI data are not available.

Our study has some limitations. First, functional MRI was not performed to confirm or compare the networks extracted from structural MRI. Second, data from the Montreal Cognitive Assessment, which is considered to be highly sensitive to MCI [49], were not available in the Iki-Iki Health Promotion Project. The MMSE is a simple and useful bedside cognitive testing tool. However, it is subjective and often insensitive to the early stages of dementia [50]. It is difficult to separate HC from individuals with MCI using cognitive function evaluation tests, which are objective indicators. In this study, MCI was diagnosed using only MMSE scores. Therefore, individuals with MCI and normal MMSE scores might have been included in the HC group [4, 51]. In contrast, participants with partial symptoms, such as prodromal AD, according to the new AD diagnostic criteria [52, 53] may have been included in the MCI group. Third, we performed SEM analysis based on the hypotheses that there are direct or indirect causal influences from the SN to the DMN and CEN [42] and that the SN plays a role in network switching between the DMN and CEN [17, 21, 42]. Brain networks influence each other in a complex manner, and the results of the SEM analysis are evaluations based on hypotheses; therefore, care must be taken when evaluating causal relationships. Fourth, our sample was restricted to relatively well-educated ethnic Japanese individuals, which may limit the generalizability of our findings to other racial and ethnic groups, particularly to those in less-developed countries. Hence, further studies are required to assess altered networks in individuals with MCI using 3 T MRI datasets from other sources, such as the Alzheimer’s Disease Neuroimaging Initiative [54]. Finally, some relevant confounders may have been excluded from the multivariate linear regression analysis.

In conclusion, our study uncovered significant disturbances within the SN among individuals with MCI. Furthermore, we discovered impairments in the switching mechanism between the CEN and the DMN, mediated by the SN. These findings suggest a critical alteration in network dynamics, highlighting the role of SN in orchestrating the complex interplay and transition of neural activities among the key brain networks in individuals with MCI. Our findings provide a deeper understanding of the underlying neural mechanisms contributing to cognitive decline.

## Abbreviations

AD: Alzheimer’s disease
CEN: Central-executive network
DMN: Default mode network
HC: Healthy controls
ICA: Independent component analysis
MCI: Mild cognitive impairment
SBM: Source-based morphometry
SEM: Structural equation modeling
SN: Salience network

## References

[1] Alzheimer’s disease facts and figures. Alzheimers Dement. 2022;18(4):700–89. 35289055 10.1002/alz.12638

[2] Eshkoor SA, Hamid TA, Mun CY, Ng CK. Mild cognitive impairment and its management in older people. Clin Interv Aging. 2015;10:687–93. 25914527 10.2147/CIA.S73922 PMC4401355

[3] Salzman T, Sarquis-Adamson Y, Son S, Montero-Odasso M, Fraser S. Associations of multidomain interventions with improvements in cognition in mild cognitive impairment: a systematic review and meta-analysis. JAMA Netw Open. 2022;5(5): e226744. 35503222 10.1001/jamanetworkopen.2022.6744 PMC9066287

[4] Petersen RC, Stevens JC, Ganguli M, Tangalos EG, Cummings JL, DeKosky ST. Practice parameter: early detection of dementia: mild cognitive impairment (an evidence-based review). Report of the Quality Standards Subcommittee of the American Academy of Neurology. Neurology. 2001;56(9):1133–42. 11342677 10.1212/wnl.56.9.1133

[5] Greicius MD, Srivastava G, Reiss AL, Menon V. Default-mode network activity distinguishes Alzheimer’s disease from healthy aging: evidence from functional MRI. Proc Natl Acad Sci U S A. 2004;101(13):4637–42. 15070770 10.1073/pnas.0308627101 PMC384799

[6] Li C, Li Y, Wu J, Wu M, Peng F, Chao Q. Triple network model-based analysis on abnormal core brain functional network dynamics in different stage of amnestic mild cognitive impairment. J Alzheimers Dis. 2022;89:519–33. 35938250 10.3233/JAD-220282

[7] Xue C, Qi W, Yuan Q, Hu G, Ge H, Rao J, et al. Disrupted dynamic functional connectivity in distinguishing subjective cognitive decline and amnestic mild cognitive impairment based on the triple-network model. Front Aging Neurosci. 2021;13: 711009. 34603006 10.3389/fnagi.2021.711009 PMC8484524

[8] Chand GB, Wu J, Hajjar I, Qiu D. Interactions of the salience network and its subsystems with the default-mode and the central-executive networks in normal aging and mild cognitive impairment. Brain Connect. 2017;7(7):401–12. 28707959 10.1089/brain.2017.0509 PMC5647507

[9] Talwar P, Kushwaha S, Chaturvedi M, Mahajan V. Systematic review of different neuroimaging correlates in mild cognitive impairment and Alzheimer’s disease. Clin Neuroradiol. 2021;31(4):953-967. 34297137 10.1007/s00062-021-01057-7

[10] Sorg C, Riedl V, Muhlau M, Calhoun VD, Eichele T, Laer L, et al. Selective changes of resting-state networks in individuals at risk for Alzheimer’s disease. Proc Natl Acad Sci U S A. 2007;104(47):18760–5. 18003904 10.1073/pnas.0708803104 PMC2141850

[11] Agosta F, Pievani M, Geroldi C, Copetti M, Frisoni GB, Filippi M. Resting state fMRI in Alzheimer’s disease: beyond the default mode network. Neurobiol Aging. 2012;33(8):1564–78. 21813210 10.1016/j.neurobiolaging.2011.06.007

[12] Zhou J, Greicius MD, Gennatas ED, Growdon ME, Jang JY, Rabinovici GD, et al. Divergent network connectivity changes in behavioural variant frontotemporal dementia and Alzheimer’s disease. Brain. 2010;133(Pt 5):1352–67. 20410145 10.1093/brain/awq075 PMC2912696

[13] Chen AC, Oathes DJ, Chang C, Bradley T, Zhou ZW, Williams LM, et al. Causal interactions between fronto-parietal central executive and default-mode networks in humans. Proc Natl Acad Sci U S A. 2013;110(49):19944–9. 24248372 10.1073/pnas.1311772110 PMC3856839

[14] Fox MD, Snyder AZ, Vincent JL, Corbetta M, Van Essen DC, Raichle ME. The human brain is intrinsically organized into dynamic, anticorrelated functional networks. Proc Natl Acad Sci U S A. 2005;102(27):9673–8. 15976020 10.1073/pnas.0504136102 PMC1157105

[15] Fransson P. Spontaneous low-frequency BOLD signal fluctuations: an fMRI investigation of the resting-state default mode of brain function hypothesis. Hum Brain Mapp. 2005;26(1):15–29. 15852468 10.1002/hbm.20113 PMC6871700

[16] Bressler SL, Menon V. Large-scale brain networks in cognition: emerging methods and principles. Trends Cogn Sci. 2010;14(6):277–90. 20493761 10.1016/j.tics.2010.04.004

[17] Chand GB, Dhamala M. Interactions among the brain default-mode, salience, and central-executive networks during perceptual decision-making of moving dots. Brain Connect. 2016;6(3):249–54. 26694702 10.1089/brain.2015.0379

[18] Menon V, Uddin LQ. Saliency, switching, attention and control: a network model of insula function. Brain Struct Funct. 2010;214(5-6):655-667. 20512370 10.1007/s00429-010-0262-0 PMC2899886

[19] Menon V. Large-scale brain networks and psychopathology: a unifying triple network model. Trends Cogn Sci. 2011;15(10):483–506. 21908230 10.1016/j.tics.2011.08.003

[20] Seeley WW, Menon V, Schatzberg AF, Keller J, Glover GH, Kenna H, et al. Dissociable intrinsic connectivity networks for salience processing and executive control. J Neurosci. 2007;27(9):2349–56. 17329432 10.1523/JNEUROSCI.5587-06.2007 PMC2680293

[21] Goulden N, Khusnulina A, Davis NJ, Bracewell RM, Bokde AL, McNulty JP, et al. The salience network is responsible for switching between the default mode network and the central executive network: replication from DCM. Neuroimage. 2014;99:180–90. 24862074 10.1016/j.neuroimage.2014.05.052

[22] Xu L, Groth KM, Pearlson G, Schretlen DJ, Calhoun VD. Source-based morphometry: the use of independent component analysis to identify gray matter differences with application to schizophrenia. Hum Brain Mapp. 2009;30(3):711–24. 18266214 10.1002/hbm.20540 PMC2751641

[23] Kakeda S, Watanabe K, Nguyen H, Katsuki A, Sugimoto K, Igata N, et al. An independent component analysis reveals brain structural networks related to TNF-α in drug-naïve, first-episode major depressive disorder: a source-based morphometric study. Transl Psychiatry. 2020;10(1):1–7. 32522975 10.1038/s41398-020-00873-8 PMC7287077

[24] Evans AC. Networks of anatomical covariance. Neuroimage. 2013;80:489–504. 23711536 10.1016/j.neuroimage.2013.05.054

[25] Ninomiya T, Nakaji S, Maeda T, Yamada M, Mimura M, Nakashima K, et al. Study design and baseline characteristics of a population-based prospective cohort study of dementia in Japan: the Japan Prospective Studies Collaboration for Aging and Dementia (JPSC-AD). Environ Health Prev Med. 2020;25(1):64. 33129280 10.1186/s12199-020-00903-3 PMC7603740

[26] Ozato N, Saitou S, Yamaguchi T, Katashima M, Misawa M, Jung S, et al. Association between visceral fat and brain structural changes or cognitive function. Brain Sci. 2021;11(8):1036. 34439655 10.3390/brainsci11081036 PMC8391376

[27] O’Bryant SE, Humphreys JD, Smith GE, Ivnik RJ, Graff-Radford NR, Petersen RC, et al. Detecting dementia with the mini-mental state examination in highly educated individuals. Arch Neurol. 2008;65(7):963–7. 18625866 10.1001/archneur.65.7.963 PMC2587038

[28] Kim H, Osuka Y, Kojima N, Sasai H, Nakamura K, Oba C, et al. Inverse association between cheese consumption and lower cognitive function in Japanese community-dwelling older adults based on a cross-sectional study. Nutrients. 2023;15:14. 10.3390/nu15143181 PMC10384548 37513598

[29] Jockusch J, Hahnel S, Nitschke I. Use of handgrip strength measurement as an alternative for assessing chewing function in people with dementia. BMC Geriatr. 2022;22(1):769. 36153477 10.1186/s12877-022-03452-2 PMC9509657

[30] Ashburner J. A fast diffeomorphic image registration algorithm. Neuroimage. 2007;38(1):95–113. 17761438 10.1016/j.neuroimage.2007.07.007

[31] Ashburner J. SPM: a history. Neuroimage. 2012;62(2):791–800. 22023741 10.1016/j.neuroimage.2011.10.025 PMC3480642

[32] Ashburner J. Computational anatomy with the SPM software. Magn Reson Imaging. 2009;27(8):1163–74. 19249168 10.1016/j.mri.2009.01.006

[33] Shattuck DW, Mirza M, Adisetiyo V, Hojatkashani C, Salamon G, Narr KL, et al. Construction of a 3D probabilistic atlas of human cortical structures. Neuroimage. 2008;39(3):1064–80. 18037310 10.1016/j.neuroimage.2007.09.031 PMC2757616

[34] Bell AJ, Sejnowski TJ. An information-maximization approach to blind separation and blind deconvolution. Neural Comput. 1995;7(6):1129–59. 7584893 10.1162/neco.1995.7.6.1129

[35] Doucet GE, Labache L, Thompson PM, Joliot M, Frangou S. Atlas55+: brain functional atlas of resting-state networks for late adulthood. Cereb Cortex. 2021;31(3):1719–31. 33188411 10.1093/cercor/bhaa321 PMC7869083

[36] Kanda Y. Investigation of the freely available easy-to-use software ‘EZR’for medical statistics. Bone Marrow Transplant. 2013;48(3):452–8. 23208313 10.1038/bmt.2012.244 PMC3590441

[37] Hu L, Bentler PM. Cutoff criteria for fit indexes in covariance structure analysis: conventional criteria versus new alternatives. Struct Equ Model. 1999;6(1):1–55.

[38] Onoda K, Ishihara M, Yamaguchi S. Decreased functional connectivity by aging is associated with cognitive decline. J Cogn Neurosci. 2012;24(11):2186–98. 22784277 10.1162/jocn_a_00269

[39] Brier MR, Thomas JB, Snyder AZ, Benzinger TL, Zhang D, Raichle ME, et al. Loss of intranetwork and internetwork resting state functional connections with Alzheimer’s disease progression. J Neurosci. 2012;32(26):8890–9. 22745490 10.1523/JNEUROSCI.5698-11.2012 PMC3458508

[40] He X, Qin W, Liu Y, Zhang X, Duan Y, Song J, et al. Abnormal salience network in normal aging and in amnestic mild cognitive impairment and Alzheimer’s disease. Hum Brain Mapp. 2014;35(7):3446–64. 24222384 10.1002/hbm.22414 PMC6869630

[41] Greicius MD, Krasnow B, Reiss AL, Menon V. Functional connectivity in the resting brain: a network analysis of the default mode hypothesis. Proc Natl Acad Sci U S A. 2003;100(1):253–8. 12506194 10.1073/pnas.0135058100 PMC140943

[42] Sridharan D, Levitin DJ, Menon V. A critical role for the right fronto-insular cortex in switching between central-executive and default-mode networks. Proc Natl Acad Sci U S A. 2008;105(34):12569–74. 18723676 10.1073/pnas.0800005105 PMC2527952

[43] Li C, Li Y, Zheng L, Zhu X, Shao B, Fan G, et al. Abnormal brain network connectivity in a triple-network model of Alzheimer’s disease. J Alzheimers Dis. 2019;69(1):237–52. 30958354 10.3233/JAD-181097

[44] John JP, Lukose A, Bagepally BS, Halahalli HN, Moily NS, Vijayakumari AA, et al. A systematic examination of brain volumetric abnormalities in recent-onset schizophrenia using voxel-based, surface-based and region-of-interest-based morphometric analyses. J Negat Results Biomed. 2015;14(1):1–15. 26065881 10.1186/s12952-015-0030-z PMC4464994

[45] Eyler LT, Elman JA, Hatton SN, Gough S, Mischel AK, Hagler DJ, et al. Resting state abnormalities of the default mode network in mild cognitive impairment: a systematic review and meta-analysis. J Alzheimers Dis. 2019;70(1):107–20. 31177210 10.3233/JAD-180847 PMC6697380

[46] Wonderlick J, Ziegler DA, Hosseini-Varnamkhasti P, Locascio J, Bakkour A, Van Der Kouwe A, et al. Reliability of MRI-derived cortical and subcortical morphometric measures: effects of pulse sequence, voxel geometry, and parallel imaging. Neuroimage. 2009;44(4):1324–33. 19038349 10.1016/j.neuroimage.2008.10.037 PMC2739882

[47] Alexander-Bloch A, Raznahan A, Bullmore E, Giedd J. The convergence of maturational change and structural covariance in human cortical networks. J Neurosci. 2013;33(7):2889–99. 23407947 10.1523/JNEUROSCI.3554-12.2013 PMC3711653

[48] Segall JM, Allen EA, Jung RE, Erhardt EB, Arja SK, Kiehl K, et al. Correspondence between structure and function in the human brain at rest. Front Neuroinform. 2012;6:10. 22470337 10.3389/fninf.2012.00010 PMC3313067

[49] Tsoi KK, Chan JY, Hirai HW, Wong SY, Kwok TC. Cognitive tests to detect dementia: a systematic review and meta-analysis. JAMA Intern Med. 2015;175(9):1450–8. 26052687 10.1001/jamainternmed.2015.2152

[50] Cai S, Huang L, Zou J, Jing L, Zhai B, Ji G, et al. Changes in thalamic connectivity in the early and late stages of amnestic mild cognitive impairment: a resting-state functional magnetic resonance study from ADNI. PLoS ONE. 2015;10(2): e0115573. 25679386 10.1371/journal.pone.0115573 PMC4332494

[51] Petersen RC. Mild cognitive impairment as a diagnostic entity. J Intern Med. 2004;256(3):183–94. 15324362 10.1111/j.1365-2796.2004.01388.x

[52] Dubois B, Feldman HH, Jacova C, Hampel H, Molinuevo JL, Blennow K, et al. Advancing research diagnostic criteria for Alzheimer’s disease: the IWG-2 criteria. Lancet Neurol. 2014;13(6):614–29. 24849862 10.1016/S1474-4422(14)70090-0

[53] Association AP. Diagnostic and statistical manual of mental disorders, fifth edition (DSM-5). 2013.

[54] Zhang J, Liu Y, Lan K, Huang X, He Y, Yang F, et al. Gray matter atrophy in amnestic mild cognitive impairment: a voxel-based meta-analysis. Front Aging Neurosci. 2021;13: 627919. 33867968 10.3389/fnagi.2021.627919 PMC8044397

